# LapEmerge trial: study protocol for a laparoscopic approach for emergency colon resection—a multicenter, open label, randomized controlled trial

**DOI:** 10.1186/s13063-024-08058-0

**Published:** 2024-04-17

**Authors:** Marie T. Grönroos-Korhonen, Jyrki A. O. Kössi

**Affiliations:** 1 Gastroenterological Surgery, Päijät-Häme Central Hospital, Keskussairaalankatu 7, 15850 Lahti, Finland; 2 Helsinki University, Helsinki, Finland

**Keywords:** Laparoscopy, Emergency surgery, Colon, Colon cancer, Large bowel obstruction, Randomized controlled trial

## Abstract

**Background:**

Due to faster recovery and lower morbidity rates, laparoscopy has become the gold standard in elective colorectal surgery for both the benign and malignant forms of the disease. A substantial proportion of colorectal operations are, however, carried out in emergency settings, and most of the emergency resections are still performed open. The aim of this study is to compare the laparoscopic versus open approach for emergency colorectal surgery.

**Method/design:**

This is a multicenter prospective randomized controlled trial including adult patients presenting with a condition requiring emergency colorectal resection.

**Discussion:**

Previous studies cautiously recommend wider use of laparoscopy in emergency colorectal resections, but all earlier reports are retrospective, are mostly single-center studies, and have limited numbers of patients. Laparoscopy may involve some unpredictable risks that have not yet been reported because of the infrequent use of the techniqueded to assess the safety of laparoscopy as well as the advantages and disadvantages of open compared with laparoscopic emergency surgery.

**Trial registration:**

Trial registration number: ClinicalTrials.gov NCT05005117. Registered on August 12, 2021.

**Supplementary Information:**

The online version contains supplementary material available at 10.1186/s13063-024-08058-0.

## Background and rationale

The first laparoscopic colon resection for colorectal cancer was performed in 1990. In the beginning, there were concerns about achieving adequate oncological results with the laparoscopic technique. Additionally, there were considerable numbers of port site metastases in the 1990s which over time diminished along with the development of laparoscopic instrumentation [1]. Initial doubts were quite soon dispelled, and laparoscopy was proven to be both safe and equal to open surgery in terms of the oncological results [2–4]. Patient recovery was also faster, postoperative ileus occurred less often, and overall morbidity was lower [4–6]. As a result, laparoscopy proved to be cost-effective [7].

Today, laparoscopic colorectal resections are well established in elective surgery, although the role of laparoscopy in emergency operations remains uncertain [8, 9]. It may be expected that the same benefits achieved with minimally invasive techniques in elective surgery also advocate the use of laparoscopy in emergency operations [10–12]. There are, however, several concerns regarding laparoscopy in emergency settings. The operations are technically more challenging due to distortion of the normal anatomy and the presence of a dilated vulnerable bowel [13]. Intra-abdominal lavage in inflammatory processes might be less effective [14] and the operation takes longer, which could be a disadvantage for the critically ill patient [15].

Approximately 10–20% of colorectal resections are carried out as emergency operations, which are associated with a higher risk of in-hospital mortality, double the risk of reoperation, and a three times higher risk of failure to rescue, compared to elective surgery [16–18]. The type of complication varies greatly depending on whether the operation has been elective or emergency, which most likely also reflects the type of technical approach. For example, fascial rupture is almost three times more common after emergency surgery [18]. In many other entities demanding emergency surgery, the laparoscopic approach has proven to diminish both morbidity and mortality [19] and can therefore be assumed to do the same in colorectal surgery.

As many as 15–20% of patients with colorectal cancer present with an obstruction [16], and it is important that the oncologic results do not get worse when a new surgical technique is employed. There are no previous reports assessing oncologic outcome between the laparoscopic and open approaches in emergency operations. When assessing the oncologic adequacy of the surgical approach, the pathology report and the quality of the surgical specimen are of the utmost importance. Classifying the surgical specimen is only possible in a prospective study design. Benz et al. proposed a new classification system for surgical specimens that facilitates this evaluation [20].

Previous reports on laparoscopy for emergency colorectal resections cautiously propose using laparoscopy more often in emergency settings, although most of the reports are retrospective, register-based or single-center studies with limited numbers of patients [21–23]. The aim of this study is to evaluate whether patients benefit from the laparoscopic approach in emergency colorectal surgery in both the benign and malignant forms of the disease.

### Objectives

The objective of this study is to evaluate whether patients benefit from the laparoscopic approach in emergency colorectal surgery, whether benign or malignant disease.

Our hypothesis is that the same beneficial effects of laparoscopy seen in elective surgery and in other entities needing emergency surgery will also apply to emergency colorectal surgery. These benefits are lower overall postoperative morbidity, shorter in-hospital stay, less postoperative ileus, less postoperative pain, fewer wound complications, and lower risk of mortality.

For oncologic patients, our hypothesis is that the laparoscopic approach is superior to the open approach, partly because of the faster recovery associated with greater access to oncologic treatment and partly because in laparoscopy the intra-operative circumstances need to be optimized to mimic the elective situation, leading to good visualization and a standardized approach.

### Trial design

**Fig. 1:**
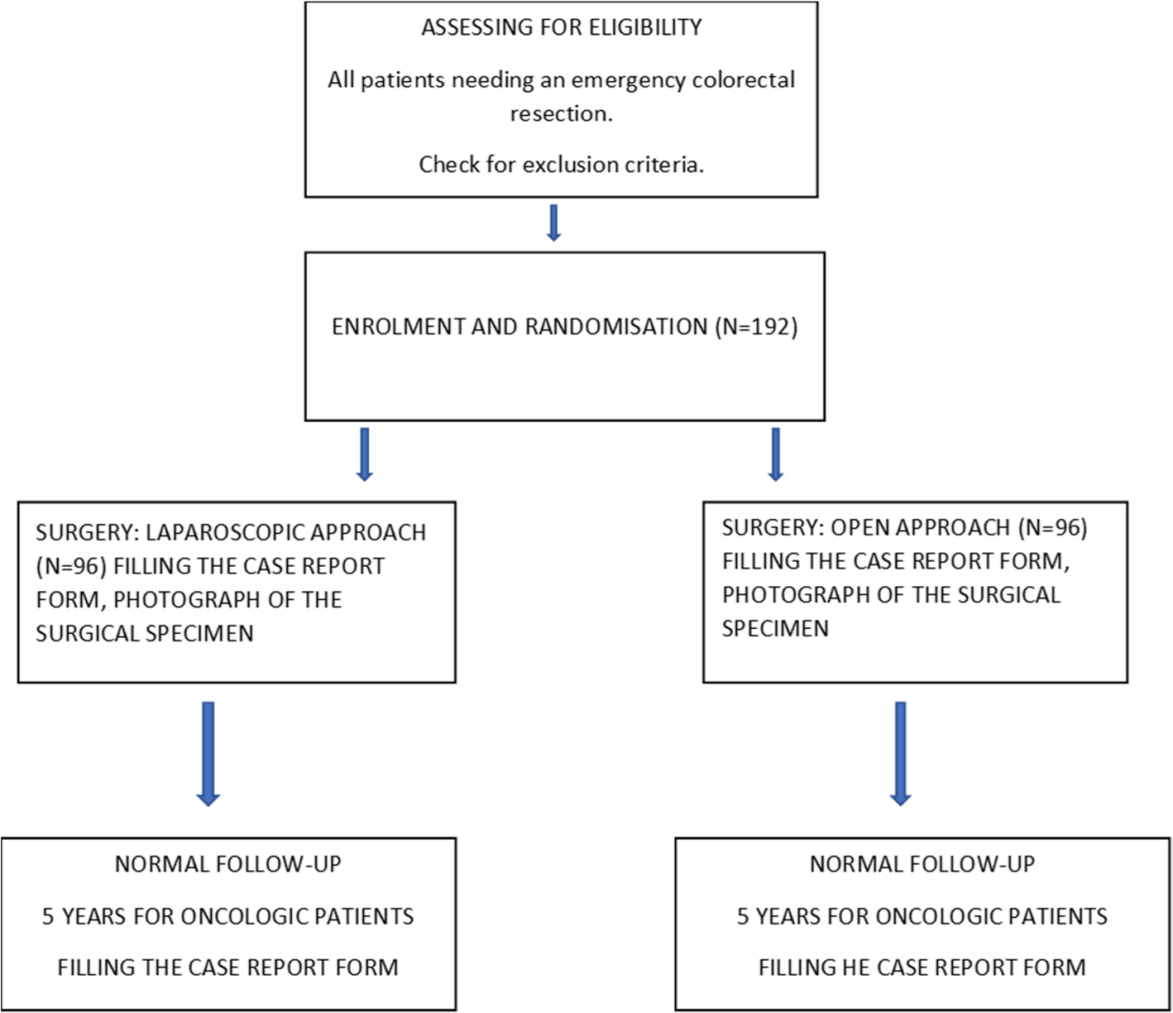
Flowchart

This study is a prospective, randomized, controlled, multicenter, superiority study comparing the laparoscopic and open approaches for emergency colorectal resections. The primary endpoint is postoperative morbidity. The trial is independent of any kind of commercial sponsorship. The flow chart of the study is presented in the (Fig. 1).

## Methods

### Study setting

This study will be carried out in six university or central hospitals in Finland: Päijät-Häme Central Hospital, Pohjois-Karjala Central Hospital, Jorvi Helsinki University Hospital, Kanta-Häme Central Hospital, Keski-Suomi Central Hospital, and Oulu University Hospital.

### Eligibility criteria

#### Inclusion criteria

The patient has a condition necessitating emergency colorectal resection. We define the timeframe for emergency colorectal resection to be within 0–48 h from the decision to proceed with surgery.

- Age 18 years or older
- The patient is competent to give their consent
- Signed informed consent

#### Exclusion criteria

- The patient’s condition is due to trauma
- Simultaneous acute pancreatitis
- The need for resection is due to a complication from a previous procedure, i.e., reoperation
- Simultaneous ruptured aortic aneurysm or ruptured aortic aneurysm as the underlying cause of the need for surgery

All patients arriving in the emergency room and diagnosed with a condition requiring emergency colorectal resection will be considered for inclusion. After receiving proper information on the possible advantages and disadvantages of the intervention, and after voluntarily signing the informed consent form, the subjects will be enrolled in the trial. The written consent will be obtained by a member of the research group and a copy given to the patient. After enrolment, the patient will be randomized for either open or laparoscopic surgery. The procedure will be carried out or closely supervised by a specialist in gastrointestinal surgery experienced in colorectal resections. Patients considered but not meeting the inclusion criteria and patients with at least one exclusion criterion will not be enrolled. These patients and the reasons for their exclusion will be documented. Postoperative care and follow-up will follow normal praxis and the case report form will be completed. Ninety days after surgery, patients will be requested to fill in the Gastrointestinal Quality of Life Index (GIQLI) questionnaire, which is the only deviation from normal follow-up. The GIQLI questionnaire will be sent by post at no cost to the patient. Once enrolled, patients will not be required to make any further visits to the hospital or to undergo any radiation or medical procedures. The follow-up for oncology patients will also be arranged according to the normal follow-up schedule.

If at any point the allocated type of intervention needs to be modified or even discontinued because of patient safety or for any other reason, the circumstances and reasons will be carefully documented and the patient will be informed. For example, if the patient’s condition changes, making the patient unfit for surgery, the patient will normally be excluded. In the event of conversion during the operation, the circumstances will be carefully documented but the patient will still be able to participate.

### Surgical technique

The operations will be conducted according to the requirements of the patient’s underlying medical condition, for example whether the reason for surgery is perforation or obstruction.

For an obstruction in the right-sided colon or transversum, the aim will be primary resection and anastomosis. The anastomosis will be done extracorporeally to avoid fecal contamination. In the case of perforation with wide contamination in the critically ill patient, an ostomy alone will be considered instead of anastomosis or with a defunctioning stoma. For an obstruction in the left-sided colon, the aim will also be to perform a primary resection with anastomosis. Because of the elevated risk of anastomotic leakage, a defunctioning loop transversostomy or ileostomy will be considered even in the absence of perforation. Depending on the clinical condition of the patient, primary resection with end colostomy is also an option.

In the case of perforation and peritonitis, intra-abdominal lavage will be performed. If the reason for surgery is colorectal cancer, the operation will be performed according to CME principles.

In laparoscopic operations, decompression of the enlarged intestine will be carried out at the beginning of the procedure by making a small incision in the site planned for specimen removal. The wound will be protected by a wound protector. After making a purse-string suture, a small enterotomy will be performed in the intestinal wall, through which a suction tube will be inserted to decompress the bowel. After sufficient decompression, the enterotomy will be closed and the lid placed on the wound protector. After this, pneumoperitoneum will be created and the procedure carried out laparoscopically according to same principles as in elective surgery. In open surgery, either a midline or a horizontal incision will be made and the procedure carried out according to normal praxis. A separate decompression is not usually needed. The wound will be closed according to Israelsson’s technique with slowly absorbable monofilament sutures.

If needed, at any point of the protocol, for patient safety or any another reason, the allocated intervention will be modified or discontinued.

### Outcomes

The main endpoint of the study will be 30-day postoperative morbidity, which will be evaluated from the Comprehensive Complication Index (CCI) of all patients. CCI is the sum of all complications weighted on with their severity based on the Clavien-Dindo classification (CCI = √(*wC* + *wC*2 + … + *wCx*)/2) and has values ranging from 0 to 100 [24].

The secondary endpoints of the study are as follows.

#### Ninety-day mortality

Ninety-day mortality is death for any reason within 90 days of surgery (yes/no).

#### Surgical site infection (SSI)

SSI is defined as infections occurring up to 30 days after surgery (or up to 1 year after surgery in patients receiving implants) and affecting either the incision or the deep tissue at the operation site. SSI is divided into superficial incisional, deep incisional, and organ/space surgical site infections [25].

#### Intensive care unit (ICU)-free days

ICU-free days are defined as the number of days during which the patient is alive up to 30 days postoperatively minus days spent in the ICU (range 0–30).

#### Length of stay (LOS)

LOS is defined as the time (days) between the index emergency operation and the day of discharge.

#### Permanent ostomy

Permanent ostomy (yes/no) is defined as no stoma reversal within 2 years of the operation, as nearly all reversals are performed within that timeframe [26].

#### Reoperation risk

This is reoperation within 30 days after primary intervention (yes/no) and the reason for reoperation. Only operations that are due to a (suspected) complication and directly related to the primary operation are considered as a reoperation.

#### Bowel function

This is the time (days) from operation to the first defecation.

#### Readmission

This is readmission within 30 days after surgery for any reason (yes/no and reason for readmission).

#### Quality of life (QoL)

QoL is measured by the GIQLI health survey [27] 90 days after surgery.

### For oncologic patients

#### Quality of surgical specimen

All surgical specimens will be photographed according to an agreed protocol. The specimen will be placed on a clean surface before the bowel is opened. The mesentery will be spread out for visualization. The main vascular branches as well as the tumor will be pointed out with an instrument (e.g., Crile). The specimen will be evaluated by an independent, blinded specialist in gastroenterological surgery. To classify a right-sided colon specimen, the Benz classification will be used [20]. Left-sided specimens will be classified according to previously agreed criteria including the level of vascular ligation and quality of mesentery (Additional file 3).

#### Pathologic report and TNM classification

- The number of lymph nodes (both the number of retrieved lymph nodes and nodes affected by cancer will be recorded)
- Site of any metastases

#### Overall survival (OS)

OS is defined as the time (years) from initial cancer treatment to death for any reason.

#### Cancer-specific survival (CSS):

CSS is defined as the time (years) from initial cancer treatment to death caused by the same cancer.

#### Disease-free survival (DFS)

DFS is defined as the time (years) from initial cancer treatment to recurrence of the cancer or death for any reason.

### Pre-intervention data

- Diagnosis and reason for the emergency operation (obstruction, perforation, ischemia etc.)
- Age, sex, BMI, ASA classification, co-morbidity according to the Charlson comorbidity index [28], previous abdominal operations, and medication use of anticoagulants, oral cortisone, or immunosuppressants
- Need for ICU treatment preoperatively (yes/no)

### Intervention data

- The surgical technique (open/laparoscopic/conversion) and type of resection using the Nordic Medico-Statistical Committee (NOMESCO) Surgical Procedural codes
- Anastomosis (intra- or extracorporeal, hand-sewn or stapled) or/and ostomy (loop-ileo-or colostomy or end ileo-or colostomy). Was the anastomosis tested intraoperatively (air–water leak test/endoscopy)?
- Contamination (preoperative/perforation/abscess/local peritonitis/general peritonitis)
- Duration of operation (min), duration of anesthesia (min), blood loss (ml), blood transfusion (IU), intraoperative fluids (ml), and the need for vasoactive drugs (noradrenaline) during the operation (yes/no)

### Post-intervention data

- Need for ICU treatment (days), need for nasogastric tube (days), flatus (days), first bowel movement (days)
- Pain medication: epidural anesthesia (days), need for opioids (days), pain medication at discharge
- LOS, discharge to home or to rehabilitation unit
- All complications described and measured by the Clavien-Dindo classification
- Demand for re-operation
- The GIQLI questionnaire (points) 90 days postoperatively
- For oncologic patients: OS, CSS, DFS, adjuvant therapy (days)
- Follow-up at 6 months, 1, 3 and 5 years
- In the case of metastatic disease: OS, CSS, chemotherapy (days)
- If remission is achieved (days)- > DFS

### Participant timeline

Recruitment of patients started in September 2021 at Päijät-Häme Central Hospital and will last for 3–4 years. The estimated enrolment schedule is presented in Table 1. The progress of enrolment is expected to vary between participating hospitals because of differences in the annual caseloads of emergency colorectal diseases. The endpoint of the study is when the final patient recruited reaches the 90-day follow-up and for the oncologic assessment when the final patient recruited reaches the 5-year follow-up or dies.

**Table 1:**
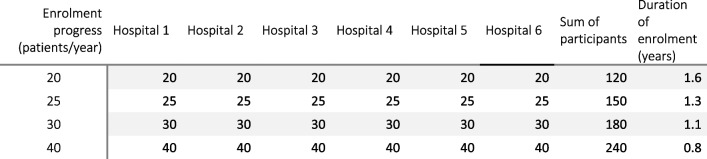
Estimate of enrolment timeline

### Sample size

Sample size calculations will be based on previous studies, which report 48.3% 30-day postoperative morbidity after open surgery and 27.8% after laparoscopy [22]. The aim of the study is to show that postoperative morbidity is lower after laparoscopic surgery. Assuming *α* = 0.05 and a (?) power = 80, 87 patients are needed for both groups. The effect size is 0.54. Assuming a 10% patient drop-out rate during the 30-day follow-up period, a total of 192 patients, i.e., 96 in each group, is needed.

### Recruitment

All subjects fulfilling the inclusion criteria will be considered for participation in the trial. An anonymous record will be kept prospectively for all subjects fulfilling the inclusion criteria but not attending, either for not consenting or for any other reason, for later assessment of selection bias. The schedule of events in the study is shown in the Table 2.

**Table 2:** SPIRIT figure

Schedule of events	Baseline	Procedure	Postoperative monitoring during hospital stay	Discharge	30 days	90 (+ 60) days	1 years	2 years	3 years	5 years	Unscheduled visit
Informed consent	X										
Need for preoperative intensive care/intensive care unit treatment	X										
Demographics and medical history	X										
Procedure details		X									
Need for postoperative intensive care/intensive care unit treatment			X								
Bowel function			X								
Analgesics			X	X							
Complications		X	X	X	X						
Gastrointestinal Quality of Life Index (GIQLI)						X					
Mortality						X					
Oncological patients: TNM classification, adjuvant chemotherapy, chemotherapy OS, CSS, DFS							X		X	X	X
Ostomy closure								X			
Protocol deviation	X	X	X	X	X		X		X	X	X

*OS* overall survival, *CSS* cancer-specific survival, *DFS* disease-free survival

## Assignment of interventions

### Allocation

Patients will be randomly allocated to the study group according to a computer-generated list, compiled by a person who is not involved in the clinical care of trial patients. The randomization will be performed in blocks of six patients. A separate randomization list will be created for each center. Another separate randomization list will be created according to the indication for surgery: obstruction, perforation, or other reason. The results of the randomization will be read by opening sealed envelopes in numerical order. Randomization will be performed after confirmation of patient eligibility and consent.

### Blinding

Blinding is impossible after surgery since wound size will reveal which technique was used. The independent surgeon evaluating the photographed surgical specimen will, however, be blinded to the technique used.

## Data collection, management, and analysis

### Data collection methods

All data will be collected prospectively into an electronic database.

The reasons for withdrawal will be carefully documented. The investigator will attempt to contact the subject at least three times prior to designating them as lost to follow-up. The investigator will document the date and type of attempted communication. If a subject cannot be reached during the visit window, a missed visit will be recorded. After three consecutive missed visits, the subject will be considered lost to follow-up, and a study exit form will be completed in the CRFs. Any data on subject participation and procedures until their withdrawal will be analyzed within the research.

### Data management

All data will be handled with the utmost confidentiality. Patients’ CRFs will be filled in by the study personnel during postoperative monitoring. The data will then be entered manually in the electronic database (SPSS). All patients will be supplied with a research number and the keyfile connecting research number with patient identification number stored separately from the main dataset. Both files will be placed into locked storage, accessed only by research personnel on faculty computers accessed only with a personal password. At the end of the study period, all data will be destroyed. Permission for the study register has been given by the ethics committee of Helsinki University Hospital and by the Päijät-Häme Central Hospital administration.

### Data monitoring

No data monitoring committee has been appointed for this trial. All complications and harmful events will be carefully reported using specific CRFs, and serious events will be reported to the principal investigator immediately. As previous studies do not provide enough information on differences in recovery time between open and laparoscopic approaches in an emergency setting, a preliminary safety analysis at 30-day follow-up on 50 patients in each group will be done. It is presumed that laparoscopy is safer, although there might be unknown factors that affect patient recovery. Any serious concern raised at any point in the study will result in its preliminary termination being considered. All authors will have access to the data. All concerns raised by any author or person outside the study group will be documented and assessed by the authors.

### Statistical methods

The findings will be analyzed using SPSS (version 27 or higher) (IBM Corp., Armonk, NY, USA) for Windows. Both intention-to-treat and per-protocol analyses will be performed.

Between-group comparisons of continuous variables will be performed using Student’s *t* test or with the Mann–Whitney *U* test if heterogeneous variances persist. Categorical data will be compared using the *χ*^2^ test or Fisher’s exact test. Long-term survival will be analyzed using Cox regression or Kaplan–Meier’s analysis. Two-tailed *p* values will be reported. *P*-values ≤ 0.05 are considered significant. If data for a certain variable is missing the case will be removed from the analysis in question.

## Ethics and dissemination

### Research ethics approval

This study follows the Declaration of Helsinki on medical protocols and ethics, and the study’s protocol has been approved by the Ethics Committee of Helsinki University Hospital 7.4.2021 (reference number 1106/2021). The amendment for participation by Pohjois-Karjala Central Hospital was approved on 30 November 2022. The amendment for participation by Keski-Suomi Central Hospital and Jorvi Helsinki University Hospital was approved on 18 January 2023. The amendment for participation by Oulu University Hospital was approved on 5 July 2023. Each participating hospital applies for study permission at their unit.

### Protocol amendments

Important protocol modifications will be communicated to the Helsinki University Hospital Ethics Committee in line with the amendments. All modifications will also be registered at ClinicalTrials.gov.

### Confidentiality

Patient confidentiality will be strictly maintained. Patients will be pseudonymized by study identification numbers, and all data will be processed without using names or personal social security numbers. Access to patient records will be limited to the study group and the study coordinator appointed by the investigator.

## Discussion

Minimally invasive surgery has become the gold standard in elective colorectal surgery because recovery is faster, less pain medication is required, postoperative morbidity is lower, and the oncological results are good [4–6, 17, 29–34]. For some reason, there has not been the same enthusiasm for minimally invasive surgery in emergency settings even though it can be presumed that the same advantages in favor of laparoscopy could well apply. Previous studies cautiously recommend wider use of laparoscopy, but as mentioned earlier, they are all retrospective, most single-center studies with small numbers of patients [17, 23, 35–41]. In retrospective studies comparing two different techniques, selection bias is inevitable. Some register-based studies have tried to balance the open and laparoscopic groups by propensity score methods [17, 23, 35–41]. However, these studies still have considerable limitations such as the retrospective nature of the study, data loss, nonuniformity, selection bias, and coding errors.

Laparoscopic emergency colorectal surgery may involve numerous possible risk factors such as iatrogenic bowel perforations, inadequately performed abdominal lavage, risk of contamination, and poor decompression. Because laparoscopy has been infrequently used in emergency situations, there might also be many unpredictable risk factors that have not yet been recognized. One theoretical disadvantage of the laparoscopic technique is the use of pneumoperitoneum in critically ill patients with peritonitis-related injury of the peritoneal lining. It has been shown that laparoscopy induces negative effects on peritoneal integrity, modifies its immune system, and induces peritoneal acidosis [42]. However, the clinical significance of these factors effects is not known.

In other words, there are probably many advantages to be gained by introducing laparoscopy more widely in emergency colorectal surgery, but at the same time the risk factors should be properly assessed.

In April 2020, Harji et al. published the LaCeS feasibility trial used to evaluate the safety and overall implementation of a prospective randomized controlled trial assessing laparoscopy versus open approach in emergency colorectal surgery. No statistical analyses were carried out, but postoperative morbidity and mortality were somewhat lower for patients who had undergone a laparoscopic procedure, indicating that laparoscopic emergency colorectal surgery could be safe. Recruitment and patient compliance were also found to be good [43]. We found the results encouraging for implementation of the LapEmerge trial, which is the first full-scale randomized, controlled study to compare open and laparoscopic emergency colorectal resections.

As a continuation of the LaCeS feasibility trial, the LaCeSS2 trial started recruiting patients in February 2022. Like the LapEmerge trial, this is also a multicenter, randomized controlled trial comparing the open and laparoscopic approaches for colon resection in emergency settings. The protocols of these trials are congruent in many ways, which will make comparison of the results not only possible but also interesting. There are also differences in focus. The LaCeS2 trial seeks to evaluate cost-effectiveness between the groups, which can be considered a strength compared to the LapEmerge trial. The target sample size is greater (512) in the LaCeS2 trial, which could also be considered a strength should it succeed but seems ambitious considering the challenges of recruitment in emergency settings and might prolong the recruitment process unnecessarily. The LapEmerge trial has a unique setting of evaluating the oncological outcome of these patients, also taking into account the quality of the surgical specimen. Since emergency colon resections for oncologic patients are inevitable and the oncologic outcome is clearly inferior to elective surgery [44], this aspect of the trial is of the utmost importance. Overall, both of these trials, which are complementary, will provide novel information on both the short- and long-term effects and safety of laparoscopy in emergency colorectal surgery.

## Trial status

Approval by the Ethics Committee of Helsinki University Hospital was received (reference1106/2021), and patient recruitment at Päijät-Häme Central Hospital started in September 2021. So far, 75 patients have been enrolled. Other hospitals started recruitment during 2023. Recruitment is expected to end on 30 September 2025. This is version 1 of the protocol.

### Supplementary Information

**Additional file 1.** Study subject information sheet.

**Additional file 2.** Consent form for a clinical research study.

**Additional file 3.** Classification of surgical specimen.

**Additional file 4.** Case report form.

## Data Availability

Marie Grönroos-Korhonen and Jyrki Kössi have access to the final trial dataset. There are no contractual agreements limiting such access for investigators.
