# Supplementary material for: LapEmerge trial: study protocol for a laparoscopic approach for emergency colon resection—a multicenter, open label, randomized controlled trial
Source: Trials. 2024 Apr 17;25:268. doi: 10.1186/s13063-024-08058-0 (PMC11022348; doi:10.1186/s13063-024-08058-0)
Supplement: Supplementary file 1 — Additional file 1. Study subject information sheet. [file 13063_2024_8058_MOESM1_ESM.docx]

**Attachment 1. Study subject information sheet.**


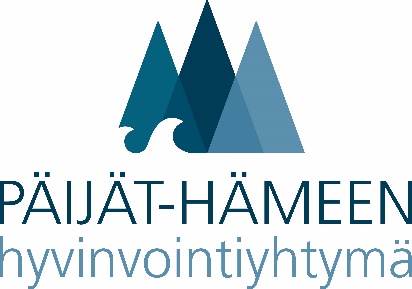


PHHYKY [Päijät-Häme Joint Authority for Health and Wellbeing]

Study subject information sheet and consent form for a clinical research study

Dear patient,

This patient information sheet describes a study in which you are being asked to participate. Before you decide whether you want to participate in the study, it is important that you read this information sheet and understand why the study is being conducted. If you have any questions, consult your doctor. If you decide to participate in the study, please sign the consent form.

Study title

Laparoscopic approach for emergency colon resection: a prospective randomized controlled trial

Invitation to participate in a study

You are invited to participate in a study investigating the differences between two surgical techniques in terms of recovery. We have assessed that you are eligible for the study, as you have been diagnosed with a condition requiring emergency colon resection. The procedure can be performed either as open surgery or by laparoscopy. Both techniques are in line with current treatment practice and knowledge. Surgery will be performed by an experienced surgeon familiar with the technique in question.

Participation is voluntary

Participation in this study is voluntary. You may decline to participate in the study, discontinue your participation or withdraw your consent at any time during the study without giving a reason and without this affecting your right to receive the treatment you need.

You do not need to participate in this study in order to receive treatment. This study will not change the treatment offered to patients with these types of conditions. The treatment procedures described in this information sheet are in line with current practice and knowledge. Your doctor can give you more information.

Take your time to read this information sheet. If you have any questions, you may contact the study doctor or other study staff (the contact details are given at the end of the document). If you decide to participate in the study, you will be asked to sign the consent form on the last page.

Party conducting the study

This national multi-centre study will be conducted by a research group led by Marie Grönroos-Korhonen Lic. Med. The contact details of the investigators in charge at your hospital are given in the consent form. The data controller of the study will be PHHYKY. Principal investigator Marie Grönroos-Korhonen will be responsible for ensuring that personal data is processed in accordance with current legislation.

Background and purpose of the study

The first laparoscopic bowel resection in the treatment of colon cancer was performed in 1990. There were worries at first regarding the safety of this new technique and long-term treatment outcomes following cancer surgery, but study results soon showed that laparoscopy was as safe as open surgery. Outcomes in the treatment of this form of cancer were also consistent. Compared with patients who had undergone open surgery, laparoscopic patients even recovered faster and had fewer bowel function problems, wound infections or troublesome pain. Today, planned bowel resections are usually performed by laparoscopy to treat both malignant and benign conditions, whereas most emergency bowel resections are still carried out by open surgery.

The aim of this prospective, randomized multi-centre study is to assess whether laparoscopy could also be beneficial in emergency bowel resections when compared to open surgery. The main aim is to investigate whether patients recover faster when they can avoid the comorbidities associated with open surgery. The study will also assess the oncological outcome in malignancies and the delay to the start of cytostatics compared with patients who’ve had open surgery. Another aim is to determine whether the duration of surgery is significantly longer in the case of laparoscopy and if it is, whether this affects patient recovery.

Study methods and study procedures

The course of the study in hospital

All adult patients arriving at the emergency clinic diagnosed with a condition requiring emergency colon resection (provided there are no exclusion criteria) will be invited to participate in the study by giving their written consent. Patients who’ve given their consent will be randomized for either open surgery or laparoscopy. In both groups, the procedure will be performed by surgeons who are experienced and also perform these operations in an elective setting. The parameters specified in the study protocol will be collected for all patients. Patients who decline to participate in the study will be treated in line with normal practice: either by open surgery or by laparoscopy, depending on the expertise of the on-call surgeon.

Surgery will be performed as required by the patient’s condition (e.g. intestinal obstruction or intestinal perforation). The aim of surgery is to perform curative resection and an intestinal anastomosis. Protective ostomy or even end ostomy may be considered, depending on the situation.

In laparoscopic surgery, bowel decompression of the enlarged intestine will be performed first. The abdominal cavity will then be filled with carbon dioxide and surgery will be continued laparoscopically. The part of the intestine to be removed will be released according to the planned surgical technique.

The surgical technique can be changed during the surgery if the patient’s condition or the situation so requires.

Open surgery will be performed using a standard procedure. The abdominal cavity will be opened in the midline or, if necessary, in a suitable location horizontally. The wound will be closed in the normal way with slowly absorbable sutures without mesh reinforcement.

Depending on their condition, patients will then be transferred to an intensive monitoring unit or a surgical ward to recover. Treatment will be individualized according to the clinical situation and the procedure in question.

After the patient is discharged, the follow-up and treatment will depend on the patient’s disease. In cancer cases, the hospital’s general treatment and follow-up practices will be followed. In benign cases, at least one follow-up visit will be scheduled to assess general recovery.

Additional samples collected during the study

The information required for the study mainly consists of the parameters necessary for the patient’s treatment, and no additional sampling will be required. Blood tests will be taken to determine values such as inflammatory values, haemoglobin, clotting factors and tumour markers. The patient’s condition will be monitored on the ward and during surgery (e.g. monitoring of blood pressure, heart rate and blood oxygen pressure). The colon that has been removed will be examined histologically, and the preparation will be photographed during surgery and the image saved in the patient’s file.

For patients with cancers, long-term outcomes will be evaluated based on information acquired from treatment-related routine check-ups. Additional appointments will not be needed during the study.

Potential benefits and potential disadvantages and discomforts attributable to the study

This study is of major clinical relevance, as it may significantly influence the treatment of patients requiring emergency colon resection. The results of the entire study will be directly applicable to patient care.

If the hypothesis of the study is confirmed and it is shown in a large prospective cohort that patients benefit from laparoscopic colon resection including in emergency situations, laparoscopy can be recommended as the primary approach for everyone.

Compared to normal practice, the study does not involve additional appointments, imaging or other study procedures that could cause harm to the patient. We’re also going to carry out an interim analysis early in the study; if it’s found at this stage that continuing the study is unethical due to significantly better results in one group, the study will be discontinued.

Data confidentiality and data protection

During the study, your identity will only be known to the study staff, all of whom are under obligation to maintain confidentiality. All data collected about you and samples collected from you will be processed in coded form, and your data cannot be identified from any results, reports or publications relating to the study.

Only such personal data that is necessary for the purpose of the study will be stored in the study register. Your name, personal identity code or contact details will not be disclosed to the study sponsor. In the study results and other documents, any references to you will only involve an identification code. The register will be stored securely in locked premises accessible only to the study doctors. In compliance with the Data Protection Act and Decree, a privacy statement has been compiled, and you can view this upon request.

With your permission/consent, data concerning your health necessary for the study may also be collected from other healthcare units and personal data records containing health data. To ensure the validity of your study data, the data will be compared, for example, with your original patient records. In this case, the data will be processed under the supervision and responsibility of the study doctor or other study staff. In all cases, your data will be processed confidentially.

If you decide to withdraw your consent, data collected up until your withdrawal will be used as part of the study material, which is necessary in order to confirm the study results.

Study expenses and reimbursement arrangements

Your treatment during the study will be provided in line with normal treatment practice. The parties to the study will not receive any other compensation.

Insurance cover for study subjects

If a procedure performed because of the study causes you a personal injury, you may apply for compensation from patient insurance. Based on the Patient Injury Act, it covers personal injuries occurring in connection with health care and medical care subject to the conditions laid down in the Act. The Finnish Patient Insurance Centre is responsible for processing compensation for patient injuries.

Conclusion of the study

The study doctor will discuss your treatment with you at check-up visits. If you wish, the study nurse will provide you with information about any publications utilizing the study results. The study doctor or the study sponsor may have to discontinue your participation prematurely. Should this happen, the measures that need to be taken will be discussed with you.

Further information

If you have any questions about the study, you can contact the study doctor or other study staff.

You can talk to them about any adverse effects that may have occurred during the study, any suspicious symptoms and other issues that worry you.

Contact details:

Marie Grönroos-Korhonen, Lic. Med., gastrosurgeon

Department of Surgery, Päijät-Häme Central Hospital

Keskussairaalankatu 7, FI-15850 Lahti

+358-444828094, [marie.gronroos-korhonen@phhyky.fi](mailto:marie.gronroos-korhonen@phhyky.fi)

**PROCESSING OF PERSONAL DATA IN THE STUDY AND THE RELATED RIGHTS OF STUDY SUBJECTS**

**Data controller**

The data controller of the study will be Päijät-Häme Central Hospital. The hospital will be responsible for ensuring that personal data is processed in accordance with the prevailing legislation.

**Processing of personal data**

Only such personal data that is necessary for the purpose of the study will be stored in the study register. Data collection will be based on the study protocol. The study subjects’ identities will only be known to the study staff, all of whom are under obligation to maintain secrecy. All data collected in the study will be processed in coded form, i.e. your name and personal identity code will be removed and replaced with a unique code. This means no subject can be identified without a decoding key. The decoding key will be stored by the person in charge of the study. Neither the study sponsor, members of the research group nor outsiders will be able to access the decoding key. The study results will be analysed in coded form.

In this study, your data will not be transferred to any party outside the EU or the European Economic Area (EEA).

The storage period of data is regulated by the prevailing legislation and Good Clinical Practice. Your personal data will be stored by PHHYKY. Your data will be stored securely for 15 years and then destroyed.

**Legal grounds for the processing of personal data**

In medical research your personal data can be processed in compliance with the Data Protection Regulation, **Article 6, Paragraph 1(e) and Article 9, Paragraph 2(i)**, when such processing is necessary for the protection of public health:

1) to determine or to assess the intended use, performance, characteristics, effects or effectiveness of the matter under investigation, or to ensure its quality, efficacy or safety; or

2) to ensure the safety of the study subjects or other persons.

In medical research your personal data can be processed in compliance with the Data Protection Regulation, **Article 6, Paragraph 1(c) and Article 9, Paragraph 2(i)**, when such processing is necessary:

1) to comply with an obligation to report adverse events or effects or with other safety reporting obligations;

2) to comply with any other study-related reporting or clarification obligations, or an obligation to store data or documents; or

3) to comply with an obligation to disclose data to the authorities.

In this study your personal data will be processed in accordance with the Data Protection Act, section 6, paragraph 2.

**Rights of the study subject**

You have the right to receive information about the processing of your personal data and to request restrictions to the processing. You also have the right to review, rectify and complete your data (e.g. if you notice an error or if the data is incomplete or unclear). You also have the right to object to the processing of your personal data. However, in the case of scientific research these rights may be subject to restrictions. The data controller may be obliged by law to store your study data for a certain period of time, in disregard of your rights. The law allows derogation from the rights of study subjects where necessary to verify the results of scientific research and to ensure the safety of the study subjects. You have the right to enquire at any time whether your personal data is being processed and, if so, to ask for the reasons. You also have the right to enquire from where your data has been obtained and to which parties your samples and data have been submitted. You have the right to receive this information free of charge and within a reasonable time (within one (1) month after your information request). If your request concerns a large amount of information or if the collection of information is particularly time-consuming for any other justifiable reason, the period may be extended by up to two (2) months. You will be notified of such extension and the grounds for it. For matters relating to data protection, please contact the person in charge of the study.

Contact details of the data controller’s data protection officer: [Add information] You have the right to lodge a complaint with a supervisory authority, in the first instance with the supervisory authority of your place of residence or place of work, if you consider that the processing of personal data infringes the EU’s General Data Protection Regulation (EU 2016/679). In Finland, this supervisory authority is the Data Protection Ombudsman.

Office of the Data Protection Ombudsman:

Lintulahdenkuja 4, FI-00530 Helsinki

PO Box 800, FI-00531 Helsinki

Switchboard: +358-29-566 6700

E-mail (registry): tietosuoja@om.fi
