# Supplementary material for: LapEmerge trial: study protocol for a laparoscopic approach for emergency colon resection—a multicenter, open label, randomized controlled trial
Source: Trials. 2024 Apr 17;25:268. doi: 10.1186/s13063-024-08058-0 (PMC11022348; doi:10.1186/s13063-024-08058-0)
Supplement: Supplementary file 2 — Additional file 2. Consent form for a clinical research study. [file 13063_2024_8058_MOESM2_ESM.docx]

**Attachment 2. Consent form for a clinical research study.**

**CONSENT TO PARTICIPATE IN A STUDY**

I have been asked to participate in the study “Laparoscopic approach for emergency colon resection; a prospective randomized controlled trial / Tähystysmenetelmä päivystyksellisissä paksunsuolen typistysleikkauksissa: etenevä, satunnaistettu tutkimus”.

I have familiarised myself with the information above and received adequate information about the study and the associated collection, processing and disclosure of data. The contents of the study have also been explained to me verbally, and I have received satisfactory answers to all my questions about the study. The information was given by _______________________________________(name of the person). I have had enough time to consider my participation in the study.

I understand that participation in this study is voluntary. I have the right to discontinue my participation in the study at any time during the study and without giving a reason. Withdrawing my consent will not cause me any negative consequences and will not affect my status as a healthcare client. I am aware that the data collected up until I withdraw my consent will be used as part of the study material.

**I hereby confirm my participation in the study described in this document and voluntarily consent to being a study subject.**

*____________________________ _______________________________*

Signature Date

*____________________________ _______________________________*

Name in block letters Date of birth or personal identity code

*__________________________________________________________*

Address

**Consent received by**

*____________________________ _______________________________*

Signature of the person receiving consent Date

*___________________________*

Name in block letters

The original signed document will remain in the study doctor’s archives, and a copy will be given to the study subject.
