# Supplementary material for: LapEmerge trial: study protocol for a laparoscopic approach for emergency colon resection—a multicenter, open label, randomized controlled trial
Source: Trials. 2024 Apr 17;25:268. doi: 10.1186/s13063-024-08058-0 (PMC11022348; doi:10.1186/s13063-024-08058-0)
Supplement: Supplementary file 3 — Additional file 3. Classification of surgical specimen. [file 13063_2024_8058_MOESM3_ESM.docx]

**Attachment 3. Classification of surgical specimen.**

**LapEmerge Case number: _________**

**Evaluation and grading of surgical specimen**

**Right-sided specimen**

Benz classification:

0 1 2 3 Points (0-3) ___________

Ungraded: Stalk of ilecolic vessels _____
 Stalk of middle colic vessels _____
 Mesenterium _____

Reason for ungrading:__________________________________

Quality of mesocolon (Benz a,b,c)

Intact mesocolon (a) Points(1) ___________

Laceration in the mesocolon: intramesocolic plane (b) Points (0.5)__________

Lacerationin the mesocolon reaching the bowel in the

vicinity of the tumor: muscularis plane. (c) Points (0) ___________

**Left-sided specimen**

Stalk of Inferior mesenteric artery (IMA) Yes____ No____ Points (1) ___________

Stalk of Inferior mesenteric vein (IMV) Yes____ No____ Points (1) ___________

Quality of mesocolon

Intact mesocolon Points (1) ___________

Laceration in the mesocolon: intramesocolic plane Points (0.75) ___________

Laceration in the mesocolon reaching the bowel in the

vicinity of the tumor: muscularis plane. Points (0.25) ___________

No mesocolon Points (0) ___________

Ungraded: Stalk of IMV ______
 Stalk of IMA ______
 Mesocolon ______
Reason for ungrading:__________________________________

**Total points:__________**
