# Supplementary material for: LapEmerge trial: study protocol for a laparoscopic approach for emergency colon resection—a multicenter, open label, randomized controlled trial
Source: Trials. 2024 Apr 17;25:268. doi: 10.1186/s13063-024-08058-0 (PMC11022348; doi:10.1186/s13063-024-08058-0)
Supplement: Supplementary file 4 — Additional file 4. Case report form. [file 13063_2024_8058_MOESM4_ESM.docx]

Attachment 4. Case report form.

Study number: ___________

Laparoscopic approach for emergent colon resection; a prospective randomized controlled trial

**PARAMETERS**

Patient has a condition requiring emergency colon resection: Yes____

Patient has had an injury: No___

It is a revision surgery: No ___

Patient has pancreatitis: No ___

Patient has underlying RAA: No ___

If you ticked all items, the patient is eligible for the study.

Diagnosis_______

Indication for emergency resection:

**Obstruction** (Yes) ______→ Colon dilated (Yes)_____ Small intestine dilated (Yes)_____

Randomize in the obstruction group.

**Perforation** (Yes) _____Masked / Local exudate _____ Abscess _____ Peritonitis____

Fecal peritonitis____

If the patient has both obstruction and perforation, randomize the patient in the perforation group.

**NB.** Abscesses (large or small / paracolic air or not) should be randomized in the group ”Other” unless the patient is critically ill and needs surgery within 0-8 hours.

**Other**, please specify (e.g. infection, ischemia, bleeding): _____________________________________

Randomize in the group ”Other”.

Study number: __________

MEDICAL HISTORY:

Age: _____

Gender: Male____ / Female ____

Height (cm): _____

Weight (kg): ______

Underlying conditions:

Prior myocardial infarction: No____ / Yes _____

Cardiac insufficiency: No____ / Yes_____

Arteriosclerosis obliterans: No____ / Yes______

Cerebral event or TIA: No_____ / Yes_____

Dementia: No_____ / Yes ______

COPD: No______/ Yes ______

Connective tissue disease: No______ / Yes ____

Gastric/duodenal ulcer: No______ / Yes ______

Liver disease: No_____ / Yes _____ → Mild / Moderate to severe (portal hypertension) _____

Diabetes: No____ / Yes ____→ End-organ complication: No___ / Yes___ Insulin: No____ / Yes ____

Hemiplegia: No___ / Yes ____

Moderate to severe kidney failure:

(Transplantation patient / dialysis / uremia / creatinine >3 mg/dl (270 **μ**mol/L): No____ / Yes ______

Malignancy: No_____ / Yes, local _____ Yes, advanced ______

Leukemia: No______ / Yes ____

Lymphoma: No______/ Yes ____

Study number: __________

AIDS: No____ / Yes____

Charlson Comorbidity Index: ______

Anticoagulation: No___ / Yes ___ Please specify: ________________

Oral cortisone: No___ / Yes____

Immunosuppression: No___ / Yes ___ Please specify: _______

Prior abdominal surgeries: _________________________________________________________

Smoker: No____ / Yes ___ Quit smoking less than a year ago: ______

Did the patient need monitoring or intensive care prior to the procedure: No_____ Yes_____

Study number: __________

**INFORMATION ABOUT THE PROCEDURE:**

ASA class: _______

Diagnosis: _______

Surgery code: ________/ Resection: ___________________(Right / extended right / left / transverse colon / subtotal / colectomy /rectum)

Technique: Open ____________ Laparoscopic _______________ Conversion _________________

If open technique, specify the incision (midline laparotomy, Kocher, transverse incision etc.): ___________

Suture:

Manual____ / Stapler____→ Linear ______ Circular ______

Intracorporeal ______ Extracorporeal______

Stoma: No_______ / Yes_______ Type: _________ Types: (1=loop colostomy/2=loop ileostomy /3=end colostomy/4=double-barrel ileocolostomy/5=end ileostomy/6=other, please specify: _________________

Start of surgery (time) (dd.mm.yyyy hh:min):

End of surgery (time):

Anesthesia prepared/ready (time):

End of anesthesia (time):

Intraoperative haemorrhage (ml):

Intraoperative red blood cell transfusion (IU): _____

Need of blood pressure support: No______ / Yes______ (ml/h, min-max) _______________

Iv fluids during surgery (ml): ________

Peroperative fecal contamination: No_____ / Yes______

Seam check: No check____ Water/Air ____ Endoscopic ____

Surgical specimen: Image_____ Dx:classification _______ SIN: Basal IMA: No_____/ Yes____ Basal MCA: No______/ Yes _____ Meso intact: No ____Yes______

Study number: __________

POSTOPERATIVE MONITORING

Intensive care within 30 days: No____ / Yes, dates: _______- _______

Intensive monitoring: No_____ / Yes, dates: _______ - ________

Nasogastric tube: No____ / Yes____ dates: ________-_________ and ____________-____________

Flatus, date: _____________

Bowel function, date: ________________

Analgesics:

Epidural: No_____/ Yes _____ Dates: ____________-______________.

Targiniq (or other long-acting opioid): No_____ / Yes____ Start date: _______ End date: _________

Short-acting opioid on the ward: No_____ / Yes____ Start date______ End date (the last date when given): ________

Analgesia at discharge: ____________________________________________

Date of discharge/transfer from the hospital where the procedure was performed: ________________

Discharge from the hospital where the procedure was performed: 0 = No, 1 = Home, 2 = health centre ward etc.

Date of discharge: ___________

Seclusion within 2 years: No______ / Yes_____ Date______ / Attempted without success, date: ________

Death: No_______ Yes______(date), Cause of death: ________________

If alive → the latest report (date): _________

Colon resection patients’ QOL survey at 3 months: No______ / Yes_______→ Score: __________

Returned to work, date: __________

Study number: __________

Complications within 30 days of surgery

Type of complication: _____________________________________

ClavienDindo grade: _____Date (start)______

Type of complication: _____________________________________

ClavienDindo grade: _____Date (start)______

Type of complication: _____________________________________

ClavienDindo grade: _____Date (start)______

Type of complication: _____________________________________

ClavienDindo grade: _____Date (start)______

Type of complication: _____________________________________

ClavienDindo grade: _____Date (start)______

Type of complication: _____________________________________

ClavienDindo grade: _____Date (start)______

Type of complication: _____________________________________

ClavienDindo grade: _____Date (start)______

The Comprehensive Complication Index (CCI): ____________________


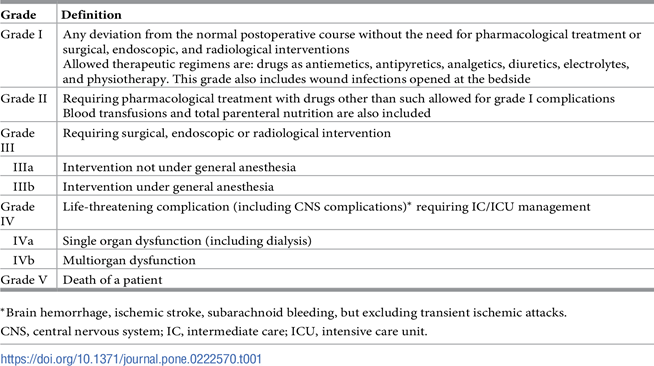


**Study number: __________**

**Oncological Oncology (?) patients:** OS (overall survival), CSS (cancer-specific survival), DFS (disease-free survival)

**The last monitoring date:** ____________

**M0 at primary stage:**

**Pathologist report, TN(?/?):_____________________________________________**

Was adjunctive treatment given: No______ / Yes______→ Start date_____

Was full adjunctive treatment given: No______ / Yes______

1 year

Relapse: No____ / Yes____ Date _____

Location of relapse_____________

Cytostatics given as treatment for relapse: No____ / Yes_____ Date ____

3 years

Relapse: No____ / Yes____ Date _____

Location of relapse _____________

Cytostatics given as treatment for relapse: No_____/ Yes_____Date ____

5 years

Relapse: No____Yes____Date _____

Location of relapse_____________

Cytostatics given as treatment for relapse: No_____Yes_____Date ____

**Primary stage M+ disease: OS, CSS**

**Pathologist report, TN(?/?)M?:____________________________________________**

Cystotatics: No____/ Yes_____Date ________

Local recurrence: No____/ Yes____Date ________

Was disease-free survival observed: No____/ Yes_____Date __________________

Was the status stable and monitored without treatment: No____ / Yes______Date _________

→ Disease relapse: No____/ Yes_____Date _________
